# Data on dynamic study of cytoophidia in *Saccharomyces cerevisiae*

**DOI:** 10.1016/j.dib.2016.05.015

**Published:** 2016-05-14

**Authors:** Hui Li, Yong Huang, Peng-Ye Wang, Fangfu Ye, Ji-Long Liu

**Affiliations:** a Medical Research Council Functional Genomics Unit, Department of Physiology, Anatomy and Genetics, University of Oxford, Oxford OX1 3PT, United Kingdom; b Key Laboratory of Soft Matter Physics, Beijing National Laboratory for Condensed Matter Physics, Institute of Physics, Chinese Academy of Sciences, Beijing 100190, China; c Key Laboratory of Entomology and Pest Control Engineering, College of Plant Protection, Southwest University, Chongqing 400715, China

**Keywords:** *Saccharomyces cerevisiae*, CTP synthase, Cytoophidium, Metabolism, Filamentation

## Abstract

The data in this paper are related to the research article entitled “Filamentation of metabolic enzymes in *Saccharomyces cerevisiae*” Q.J. Shen et al. (2016) [1]. Cytoophidia are filamentous structures discovered in fruit flies (doi:10.1016/S1673-8527(09)60046-1) J.L. Liu (2010) [2], bacteria (doi:10.1038/ncb2087) M. Ingerson-Mahar et al. (2010) [3], yeast (doi:10.1083/jcb.201003001; doi:10.1242/bio.20149613) C. Noree et al. (2010) and J. Zhang, L. Hulme, J.L. Liu (2014) [4], [5] and human cells (doi:10.1371/journal.pone.0029690; doi:10.1016/j.jgg.2011.08.004) K. Chen et al. (2011) and W.C. Carcamo et al. (2011) ( [6], [7]. However, there is little research on the motility of the cytoophidia. Here we selected cytoophidia formed by 6 filament-forming proteins in the budding yeast *S*. *cerevisiae*, and performed living-cell imaging of cells expressing the proteins fused with GFP. The dynamic features of the six types of cytoophidia were analyzed. In the data, both raw movies and analysed results of the dynamics of cytoophidia are presented.

**Specifications Table**

**Table:** 

Subject area	*Biology*
More specific subject area	*Genetics and Molecular Biology*
Type of data	*Movie and figure*
How data was acquired	*Confocal Microscope*
Data format	*Analyzed*
Experimental factors	*Movies were collected at room temperature with the speed 1.29 s/frame. The pixel size was 0.162 µm.*
Experimental features	*For the imaging, the S.pombe cells were pipetted onto Glass Bottom Dishes at stationary stage with PBS dilution. For the data processes, the MSD analysis of the trajectories were performed using Matlab.*
Data source location	*University of Oxford*
Data accessibility	*Data are with this article*

**Value of the data**

• Raw movies of cytoophidia in budding yeast are shared, which can be directly used for analysis with broad purposes. The cytoophidia includes Acc1p (acetyl-CoA carboxylase), Asn1p (asparagine requiring), Gcd2p (General Control Derepressed), Gdb1p (glycogen debranching enzyme), Glt1p (Glutamate synthase) and Pfk2p (Phosphofructokinase).
• Dynamic feature of the above 6 kinds of cytoophidia are shown separately, which can be useful to be compared with other results.
• We provide an effective method for tracking and analyzing the cytoophidia in budding yeast.

## 1 Data

**Fig. 1:**
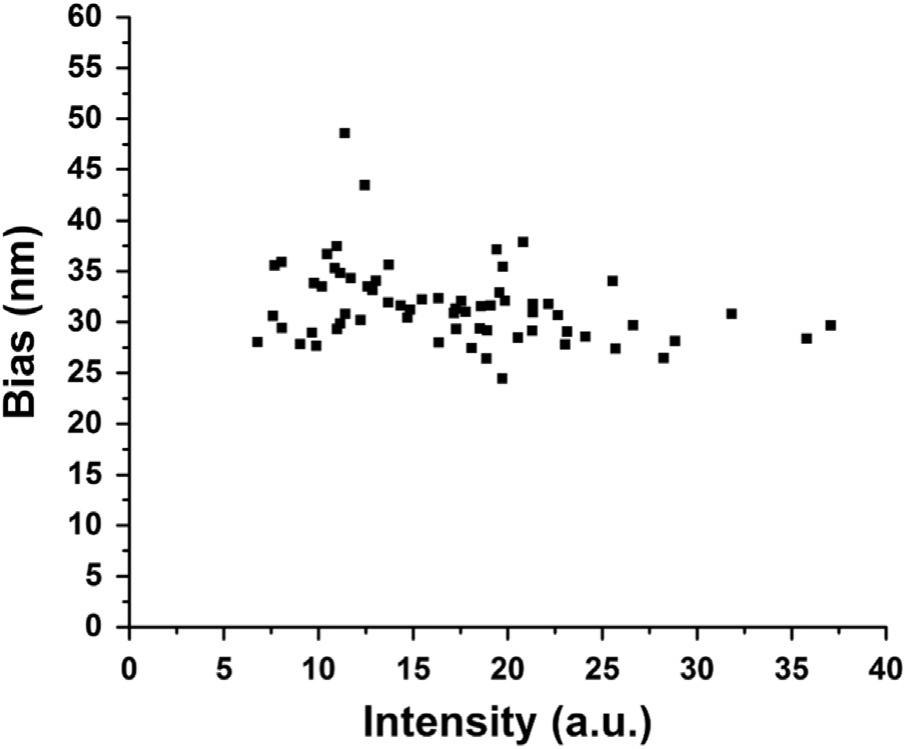
Location accuracy for the cytoophidia in fixed budding yeast. The cytoophidia were immobile in the PFA fixed budding yeast. The mean bias of repetitive localization of total 66 cytoophidia is about 31.6 nm. The intensity of cytoophidia is correlated with the size of filaments.

**Fig. 2:**
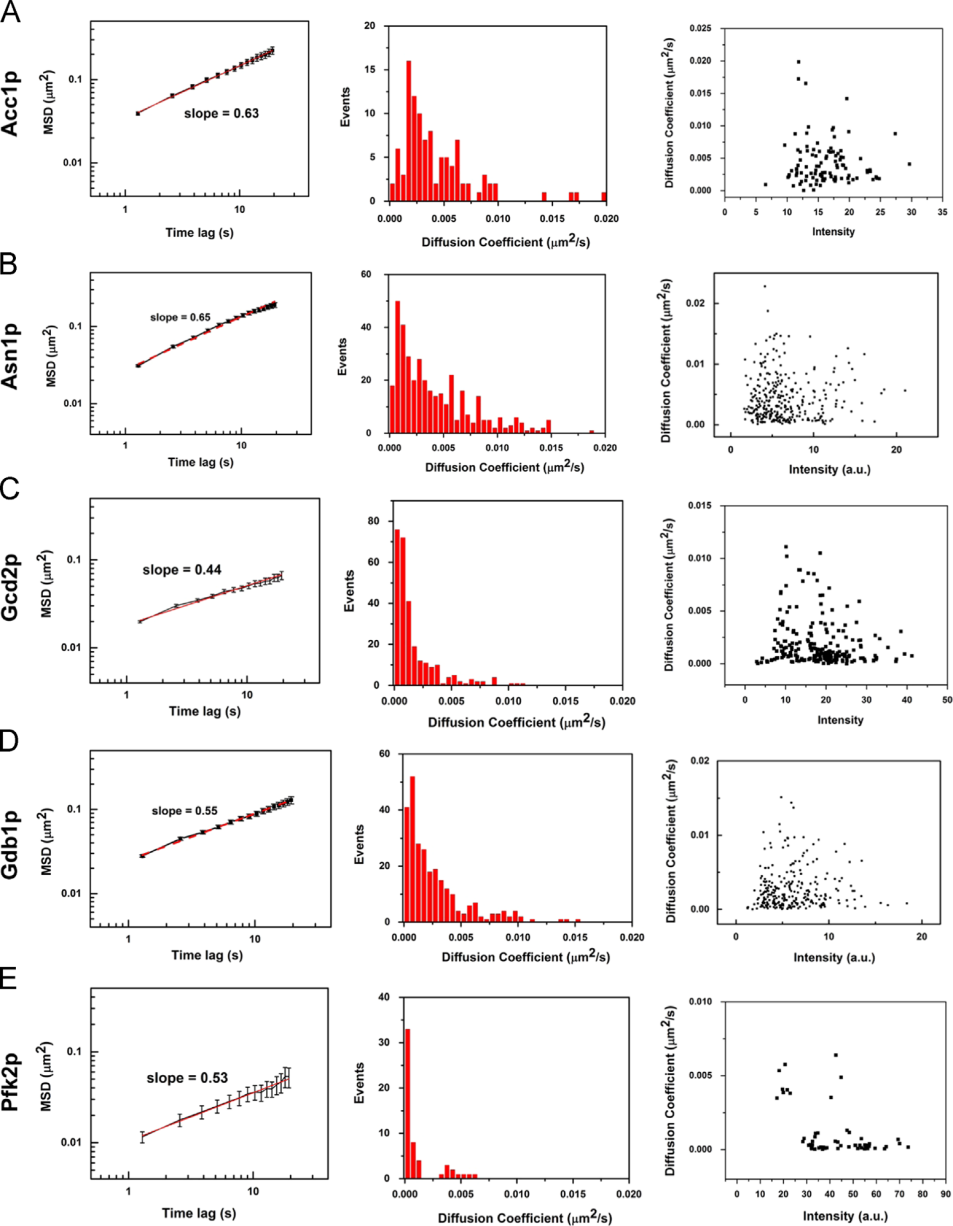
Summary of the dynamic analysis for 5 kinds of cytoophidia in budding yeast: Acc1p (A), Asn1p (B), Gcd2p (C), Gdb1p (D) and Pfk2p (E). The left panel is the average MSD as a function of time lag plotted in log-log scale. The slope of MSD here represents the exponent *ɑ*. The middle panel is the distribution of diffusion coefficients for the trajectories. The right panel is the diffusion coefficients versus the fluorescence intensities of cytoophidia. Trajectory number for each group: Acc1p (*n*=122), Asn1p (*n*=376), Gcd2p (*n*=289), Gdb1p (*n*=266), and Pfk2p (*n*=64).

In the data, Fig. 1 shows the location accuracy for the cytoophidia in fixed budding yeast, demonstrating that our tracking method is suitable for the foci and filament of cytoophidia. Fig. 2 is the summary of the dynamic analysis for 5 kinds of cytoophidia: Acc1p, Asn1p, Gcd2p, Gdb1p, and Pfk2p. Movie 1–6 are the raw videos of budding yeast expressing 6 kinds of GFP-fused proteins respectively. Movie 7–8 show the typically movements of the fast and slow Glt1p cytoophidia respectively.

Supplementary material related to this article can be found online at doi:10.1016/j.dib.2016.05.015.

The following is the Supplementary material related to this article Movie 1, Movie 2, Movie 3, Movie 4, Movie 5, Movie 6, Movie 7, Movie 8.

Movie 1 Time lapse of *Saccharomyces cerevisiae* cells expressing Acc1p-GFP. Duration: 10 min; recorded at 1.29 s/frame. The original movie was cropped and compressed to reduce the file size for publish.

Movie 2 Time lapse of *S*. *cerevisiae* cells expressing Asn1p-GFP. Duration: 10 min; recorded at 1.29 s/frame. The original movie was cropped and compressed to reduce the file size for publish.

Movie 3 Time lapse of *S*. *cerevisiae* cells expressing Gcd2p-GFP. Duration: 10 min; recorded at 1.29 s/frame. The original movie was cropped and compressed to reduce the file size for publish.

Movie 4 Time lapse of *S*. *cerevisiae* cells expressing Gdb1p-GFP. Duration: 10 min; recorded at 1.29 s/frame. The original movie was cropped and compressed to reduce the file size for publish.

Movie 5 Time lapse of *S*. *cerevisiae* cells expressing Glt1p-GFP. Duration: 10 min; recorded at 1.29 s/frame. The original movie was cropped and compressed to reduce the file size for publish.

Movie 6 Time lapse of *S*. *cerevisiae* cells expressing Pfk2p-GFP. Duration: 10 minute; recorded at 1.29 s/frame. The original movie was cropped and compressed to reduce the file size for publish.

Movie 7 Movie of the Glt1p cytoophidium diffusing fast. The original movie was recorded at 1.29 s/frame, and this movie is played at 2 frames/s. The tracking was performed as described in the text, and the trajectory plot was produced using the ImageJ plugin Particle Tracker.

Movie 8 Movie of the Glt1p cytoophidium diffusing slowly. The original movie was recorded at 1.29 s/frame. The number of frames were reduced for a smaller file size and the movie is played at 2 frames/s. The process method was same as in Movie S7.

## 2 Experimental design, materials and methods

### 2.1 Live cell imaging

*S*. *cerevisiae* strains used here were derived from the yeast GFP clone collection comprising of 4159 GFP tagged open reading frames (ORF) [8]. All strains were shaken at 32 °C for 24 h before the live cell imaging. Cells at stationary stage were pipetted onto Glass Bottom Dishes (MatTek Corporation, Ashland, MA) with appropriate dilution in PBS. The cells dispersed in a single layer on the bottom. After 30 min incubating at room temperature, the cells were imaged using the 63X objective on a laser-scanning confocal microscope (Leica TCS SP5 II confocal microscope). Time-lapse videos were taken at 1.29 s intervals between each frame over 10 min of real time. All the videos were captured at room temperature. At least 3 videos for each sample were taken with random fields containing filaments.

For the immobilized cytoophidia, cells were fixed in 4% paraformaldehyde (PFA) for 10 min, and then washed by PBS.

### 2.2 Tracking

Tracking of the cytoophidia was performed by using the ImageJ plugin Particle Tracker [9]. The effectiveness of the software for tracking the filament-formed cytoophidia was proved. In each frame, the cytoophidia were localized first. The greatest number of clearly visible cytoophidia were need to be captured. Generally, the parameter radius was set to 5–7 pixels and the percentile was set to 0.3–0.5% in Particle Tracker software. In the linking step, the parameters of linking range and displacement were set to 2 frames and 5 pixels respectively. All the linking parameters for each kind of cytoophidia were optically checked. The trajectories were saved in txt format for further analysis.

### 2.3 Dynamic analysis

Only the trajectories longer than 15 frames (approximately 20 s) were selected. The MSD of each trajectory was calculated by the equation

(1) $$\text{MSD}(\tau )\;=\;\langle {\left|r(t+\tau )-r(t)\right|}^{2}\rangle$$

where *τ* is the time lag. Then the first 15 points of MSD were fitted by the power law equation

(2) $$\text{MSD}(\tau )\;=\;\text{A}\cdot \tau^{\alpha }$$

The exponent *α* indicates the non-linear relationship of MSD with time, carrying information about the motion type of the diffusion [10]: *ɑ*~1 corresponds to free Brownian motion (i.e. free diffusion), *α*<1 sub-diffusion (i.e. diffusion within a crowded medium), *α*>1 super-diffusion (i.e. diffusion overlaid wi**t**h directed motion). Similarly, the diffusion coefficient *D* is determined by fitting the 3 initial points of the MSD curves with

(3) MSD(τ)=4D⋅τ+c

All the analyses were performed using a user-defined program in Matlab.

## Footnotes

Appendix A Supplementary data associated with this article can be found in the online version at doi:10.1016/j.dib.2016.05.015.

## References

[1] Shen Q.J. (2016). Filamentation of metabolic enzymes in *Saccharomyces cerevisiae*. J. Genet. Genom..

[2] Liu J.L. (2010). Intracellular compartmentation of CTP synthase in Drosophila. J. Genet. Genom..

[3] Ingerson-Mahar M. (2010). The metabolic enzyme CTP synthase forms cytoskeletal filaments. Nat. Cell. Biol..

[4] Noree C. (2010). Identification of novel filament-forming proteins in *Saccharomyces cerevisiae* and *Drosophila melanogaster*. J. Cell. Biol..

[5] Zhang J., Hulme L., Liu J.L. (2014). Asymmetric inheritance of cytoophidia in *Schizosaccharomyces pombe*. Biol. Open.

[6] Chen K. (2011). Glutamine analogs promote cytoophidium assembly in human and Drosophila cells. J. Genet. Genom..

[7] Carcamo W.C. (2011). Induction of cytoplasmic rods and rings structures by inhibition of the CTP and GTP synthetic pathway in mammalian cells. Plos One.

[8] Huh W.K. (2003). Global analysis of protein localization in budding yeast. Nature.

[9] Sbalzarini I.F., Koumoutsakos P. (2005). Feature point tracking and trajectory analysis for video imaging in cell biology. J. Struct. Biol..

[10] Saxton M.J., Jacobson K. (1997). Single-particle tracking: applications to membrane dynamics. Annu. Rev. Biophys. Biomol. Struct..

