# Supplementary material for: Data on dynamic study of cytoophidia in Saccharomyces cerevisiae
Source: Data Brief. 2016 May 14;8:40–4. doi: 10.1016/j.dib.2016.05.015 (PMC4885114; doi:10.1016/j.dib.2016.05.015)
Supplement: Supplementary file 9 — Supplementary material [file mmc1.docx]

**DIB-D-16-00244R1**

We declare there is no conflict of interest.
